# Supplementary material for: A Likelihood Approach for Real-Time Calibration of Stochastic Compartmental Epidemic Models
Source: PLoS Comput Biol. 2017 Jan 17;13(1):e1005257. doi: 10.1371/journal.pcbi.1005257 (PMC5240920; doi:10.1371/journal.pcbi.1005257)
Supplement: S1 File — (TAR.GZ) [file pcbi.1005257.s014.tar.gz › HSPH_Online-SI-Revision/EnKF07/n10-severe/n10-severe_EnKF2_table0.pdf]

1	newly 3 weeks spec	422.	26.05	{0., 91.}	93.827	2.74117	newly 3 weeks cumul	1572.	1686.86	{45., 7622.}	131.982	0.779486	newly 5 weeks spec	319.	19.02	{0., 63.}	94.0376	2.67575	newly 5 weeks cumul	2230.	1728.38	{59., 7622.}	108.817	0.82237	newly 51 weeks cumul	3236.	1868.34	{91., 7622.}	90.7064	0.839386
2	newly 3 weeks spec	559.	106.54	{13., 186.}	82.0107	0.969864	newly 3 weeks cumul	1850.	544.05	{147., 1154.}	72.8665	0.681171	newly 5 weeks spec	307.	54.71	{0., 150.}	82.1792	1.33592	newly 5 weeks cumul	2596.	672.93	{185., 1261.}	75.3417	0.731966	newly 53 weeks cumul	3551.	919.12	{200., 2004.}	76.33	0.779422
3	newly 3 weeks spec	372.	131.55	{0., 111.}	110.567	2.03116	newly 3 weeks cumul	1462.	1137.91	{47., 3451.}	111.124	0.802186	newly 5 weeks spec	176.	66.31	{0., 91.}	109.483	1.90718	newly 5 weeks cumul	1920.	1299.71	{57., 3451.}	106.191	0.834166	newly 55 weeks cumul	2258.	1449.32	{66., 3451.}	102.752	0.836435
4	newly 3 weeks spec	431.	275.3	{4., 975.}	120.097	1.16953	newly 3 weeks cumul	1668.	1237.42	{69., 5243.}	109.603	0.791179	newly 5 weeks spec	272.	92.71	{0., 128.}	106.033	1.8149	newly 5 weeks cumul	2316.	1489.8	{78., 7320.}	105.476	0.868378	newly 50 weeks cumul	2990.	1594.82	{81., 7889.}	100.167	0.940143
5	newly 3 weeks spec	395.	110.86	{0., 160.}	97.6658	1.5431	newly 3 weeks cumul	1512.	2160.97	{151., 9334.}	153.785	0.630814	newly 5 weeks spec	274.	5.31	{0., 11.}	98.062	2.80632	newly 5 weeks cumul	2125.	2189.33	{151., 9651.}	129.149	0.724469	newly 50 weeks cumul	3018.	2191.63	{151., 9662.}	110.469	0.824341
6	newly 3 weeks spec	500.	170.82	{0., 524.}	97.656	2.01883	newly 3 weeks cumul	1731.	1154.88	{44., 4582.}	111.221	0.975069	newly 5 weeks spec	216.	81.93	{0., 44.}	114.958	2.19635	newly 5 weeks cumul	2283.	1359.34	{48., 5143.}	106.607	1.04688	newly 51 weeks cumul	2710.	1459.67	{48., 6383.}	102.902	1.09324
7	newly 3 weeks spec	266.	283.62	{5., 1049.}	152.06	1.03684	newly 3 weeks cumul	943.	1156.69	{112., 4218.}	144.472	0.644678	newly 5 weeks spec	142.	128.99	{0., 427.}	145.514	1.54114	newly 5 weeks cumul	1279.	1466.21	{131., 4891.}	144.517	0.727058	newly 50 weeks cumul	1805.	1741.94	{148., 8031.}	135.563	0.82716
8	newly 3 weeks spec	348.	159.78	{14., 148.}	95.569	0.786798	newly 3 weeks cumul	1582.	577.42	{53., 490.}	92.4273	0.806183	newly 5 weeks spec	203.	101.25	{3., 140.}	93.3153	0.877617	newly 5 weeks cumul	2074.	806.61	{69., 786.}	91.9465	0.792535	newly 54 weeks cumul	2481.	1374.	{103., 2799.}	88.1806	0.713126
9	newly 3 weeks spec	571.	70.39	{7., 119.}	88.4711	1.21872	newly 3 weeks cumul	2047.	401.7	{37., 579.}	84.3322	0.991985	newly 5 weeks spec	361.	27.87	{1., 57.}	92.2798	1.52289	newly 5 weeks cumul	2908.	473.2	{51., 659.}	85.5619	1.05874	newly 58 weeks cumul	3573.	548.8	{70., 777.}	85.4123	1.07376
10	newly 3 weeks spec	479.	76.39	{2., 125.}	85.1002	1.16371	newly 3 weeks cumul	1759.	436.65	{28., 619.}	80.9682	0.933244	newly 5 weeks spec	209.	34.27	{0., 61.}	89.5646	1.35497	newly 5 weeks cumul	2339.	520.65	{33., 725.}	80.9649	0.97476	newly 58 weeks cumul	2666.	604.76	{33., 829.}	80.877	0.971916
11	newly 3 weeks spec	358.	36.59	{0., 86.}	93.1704	2.12799	newly 3 weeks cumul	1919.	1225.43	{64., 3374.}	107.842	0.857231	newly 5 weeks spec	90.	24.9	{0., 53.}	94.3556	1.99611	newly 5 weeks cumul	2152.	1278.47	{72., 3450.}	103.704	0.843661	newly 57 weeks cumul	2254.	1404.06	{87., 3450.}	101.681	0.788911
12	newly 3 weeks spec	587.	45.15	{0., 97.}	93.937	2.2902	newly 3 weeks cumul	1997.	1324.73	{28., 3284.}	109.395	0.910515	newly 5 weeks spec	284.	31.13	{0., 71.}	94.9824	2.41115	newly 5 weeks cumul	2683.	1391.98	{38., 3343.}	99.3157	0.945396	newly 57 weeks cumul	3203.	1561.12	{48., 4071.}	93.744	0.917599
13	newly 3 weeks spec	574.	179.13	{14., 214.}	94.4164	0.980015	newly 3 weeks cumul	2148.	657.66	{55., 687.}	90.6881	0.899211	newly 5 weeks spec	276.	119.85	{4., 140.}	99.2428	1.02597	newly 5 weeks cumul	2826.	921.73	{80., 1140.}	90.9565	0.8943	newly 54 weeks cumul	3484.	1586.87	{148., 2003.}	94.8608	0.859995
14	newly 3 weeks spec	353.	193.08	{22., 316.}	95.4788	0.767211	newly 3 weeks cumul	1439.	1201.55	{252., 1799.}	102.301	0.546579	newly 5 weeks spec	163.	81.54	{0., 73.}	111.08	1.17751	newly 5 weeks cumul	1856.	1406.68	{278., 2027.}	101.089	0.595351	newly 56 weeks cumul	2090.	1532.41	{286., 2146.}	101.157	0.615864
15	newly 3 weeks spec	511.	194.59	{2., 750.}	96.045	1.34871	newly 3 weeks cumul	2278.	1994.01	{116., 8574.}	116.729	0.795686	newly 5 weeks spec	197.	39.96	{0., 121.}	88.9543	1.92452	newly 5 weeks cumul	2829.	2116.5	{119., 9047.}	107.61	0.834674	newly 60 weeks cumul	3055.	2189.85	{119., 9170.}	104.297	0.844859
16	newly 3 weeks spec	366.	149.62	{16., 331.}	91.8087	0.851581	newly 3 weeks cumul	1694.	1018.45	{229., 1463.}	94.3371	0.630494	newly 5 weeks spec	184.	46.54	{0., 56.}	95.75	1.34266	newly 5 weeks cumul	2137.	1145.87	{247., 1881.}	92.9083	0.676267	newly 56 weeks cumul	2454.	1218.7	{247., 2599.}	91.3431	0.706108
17	newly 3 weeks spec	538.	177.76	{22., 465.}	88.7212	0.909934	newly 3 weeks cumul	1689.	1040.44	{212., 981.}	96.4618	0.631468	newly 5 weeks spec	353.	114.71	{2., 80.}	106.535	1.4144	newly 5 weeks cumul	2470.	1294.26	{238., 3579.}	94.7174	0.716888	newly 56 weeks cumul	3959.	1533.42	{245., 4187.}	91.8727	0.850535
18	newly 3 weeks spec	567.	135.2	{18., 122.}	92.8254	1.05521	newly 3 weeks cumul	1753.	523.05	{126., 489.}	90.9646	0.900684	newly 5 weeks spec	394.	82.61	{3., 93.}	92.4645	1.22963	newly 5 weeks cumul	2605.	710.54	{230., 615.}	90.6718	0.938076	newly 54 weeks cumul	3791.	1122.44	{249., 1431.}	89.3242	0.939473
19	newly 3 weeks spec	442.	134.19	{1., 195.}	100.26	1.55617	newly 3 weeks cumul	1574.	2470.58	{232., 9953.}	156.632	0.585881	newly 5 weeks spec	313.	9.82	{0., 26.}	96.8626	2.8275	newly 5 weeks cumul	2213.	2516.59	{234., 9960.}	128.919	0.666532	newly 50 weeks cumul	2944.	2520.69	{260., 9960.}	112.063	0.740598
20	newly 3 weeks spec	441.	108.93	{6., 254.}	91.5533	1.29434	newly 3 weeks cumul	1300.	1186.19	{95., 1479.}	132.642	0.826464	newly 5 weeks spec	373.	21.07	{0., 43.}	97.1823	2.19837	newly 5 weeks cumul	2074.	1244.8	{101., 1653.}	112.886	0.961905	newly 50 weeks cumul	3655.	1287.94	{101., 2394.}	99.4331	1.15629
21	newly 3 weeks spec	460.	71.16	{4., 135.}	85.2739	1.13969	newly 3 weeks cumul	1920.	415.01	{23., 625.}	83.3682	1.0018	newly 5 weeks spec	202.	32.92	{0., 59.}	88.3267	1.36218	newly 5 weeks cumul	2437.	494.27	{30., 726.}	82.5462	1.01811	newly 58 weeks cumul	2952.	573.53	{42., 858.}	82.211	1.02886
22	newly 3 weeks spec	509.	199.08	{16., 387.}	93.8468	0.927041	newly 3 weeks cumul	1513.	1077.36	{213., 1874.}	98.8473	0.597244	newly 5 weeks spec	221.	99.99	{2., 89.}	110.701	1.2236	newly 5 weeks cumul	2121.	1322.08	{235., 2453.}	99.0486	0.674999	newly 56 weeks cumul	2643.	1476.7	{239., 2621.}	97.2493	0.722282
23	newly 3 weeks spec	421.	119.42	{0., 104.}	104.665	2.08912	newly 3 weeks cumul	1389.	1131.16	{39., 3770.}	114.665	0.828324	newly 5 weeks spec	276.	60.48	{0., 89.}	99.6957	2.07379	newly 5 weeks cumul	2005.	1277.17	{45., 3770.}	104.601	0.879893	newly 55 weeks cumul	2915.	1426.07	{54., 3770.}	96.4456	0.936841
24	newly 3 weeks spec	587.	167.85	{22., 217.}	90.7581	0.959213	newly 3 weeks cumul	2481.	808.41	{217., 1165.}	80.5728	0.732399	newly 5 weeks spec	276.	63.78	{1., 79.}	97.1159	1.31629	newly 5 weeks cumul	3169.	973.76	{237., 1369.}	83.2799	0.784684	newly 56 weeks cumul	3630.	1070.15	{245., 1427.}	84.7044	0.815224
25	newly 3 weeks spec	500.	145.07	{3., 464.}	89.602	1.29522	newly 3 weeks cumul	2503.	1456.02	{146., 7710.}	100.663	0.818325	newly 5 weeks spec	93.	32.35	{0., 63.}	101.946	1.80769	newly 5 weeks cumul	2800.	1553.39	{158., 7768.}	98.7832	0.829442	newly 59 weeks cumul	2905.	1609.82	{168., 7768.}	98.4957	0.829432
26	newly 3 weeks spec	372.	81.86	{11., 117.}	84.0968	0.978108	newly 3 weeks cumul	1508.	470.26	{53., 597.}	80.7997	0.805966	newly 5 weeks spec	264.	34.3	{1., 51.}	91.1667	1.42975	newly 5 weeks cumul	2071.	556.33	{66., 696.}	82.1096	0.86471	newly 58 weeks cumul	2508.	652.79	{70., 778.}	81.9454	0.886576
27	newly 3 weeks spec	638.	119.87	{0., 126.}	96.0517	1.873	newly 3 weeks cumul	2129.	771.94	{48., 751.}	99.0991	1.06582	newly 5 weeks spec	370.	38.14	{0., 47.}	95.9135	2.48473	newly 5 weeks cumul	2994.	873.31	{51., 1178.}	95.7211	1.16149	newly 59 weeks cumul	3855.	979.42	{52., 1178.}	94.3471	1.24294
28	newly 3 weeks spec	519.	73.56	{5., 135.}	86.8593	1.17846	newly 3 weeks cumul	1948.	412.01	{32., 610.}	83.4143	0.990504	newly 5 weeks spec	219.	31.33	{1., 67.}	86.6895	1.31158	newly 5 weeks cumul	2468.	491.15	{39., 734.}	83.0255	1.00539	newly 58 weeks cumul	2735.	561.41	{52., 910.}	81.8534	0.980051
29	newly 3 weeks spec	430.	49.02	{4., 78.}	93.0791	1.41956	newly 3 weeks cumul	2046.	515.07	{162., 1286.}	84.4384	0.918452	newly 5 weeks spec	157.	23.77	{0., 13.}	103.599	2.20643	newly 5 weeks cumul	2479.	573.44	{167., 1536.}	86.2816	0.978556	newly 60 weeks cumul	2601.	610.26	{167., 1691.}	86.875	0.98987
30	newly 3 weeks spec	581.	374.81	{2., 1455.}	124.363	1.49226	newly 3 weeks cumul	2678.	1038.97	{25., 4929.}	90.3536	1.10343	newly 5 weeks spec	176.	106.37	{0., 526.}	122.892	2.07455	newly 5 weeks cumul	3250.	1361.37	{29., 6129.}	93.7929	1.15352	newly 60 weeks cumul	3417.	1466.91	{29., 8303.}	95.0609	1.16715
31	newly 3 weeks spec	369.	35.92	{0., 74.}	91.5122	2.01889	newly 3 weeks cumul	1285.	988.2	{95., 3711.}	119.689	0.769778	newly 5 weeks spec	236.	24.83	{0., 55.}	94.3771	2.32074	newly 5 weeks cumul	1779.	1040.67	{119., 3711.}	106.367	0.820276	newly 57 weeks cumul	2329.	1197.42	{119., 3811.}	97.9227	0.821775
32	newly 3 weeks spec	297.	305.12	{10., 1369.}	140.013	0.942323	newly 3 weeks cumul	969.	1362.46	{108., 3587.}	143.686	0.608443	newly 5 weeks spec	208.	111.6	{0., 162.}	116.385	1.54401	newly 5 weeks cumul	1426.	1651.53	{121., 5906.}	134.357	0.708577	newly 50 weeks cumul	2189.	1812.46	{123., 8317.}	116.242	0.826115
33	newly 3 weeks spec	452.	363.21	{10., 1232.}	113.794	0.957486	newly 3 weeks cumul	1482.	1828.86	{337., 5614.}	114.742	0.499827	newly 5 weeks spec	292.	106.95	{0., 275.}	93.9418	1.68764	newly 5 weeks cumul	2155.	2136.42	{422., 7457.}	105.798	0.575887	newly 50 weeks cumul	3095.	2262.13	{443., 7752.}	92.7913	0.654489
34	newly 3 weeks spec	392.	159.91	{0., 655.}	109.819	2.04486	newly 3 weeks cumul	1312.	1672.55	{156., 7582.}	158.69	0.780307	newly 5 weeks spec	229.	36.72	{0., 54.}	99.214	2.73272	newly 5 weeks cumul	1839.	1780.34	{156., 8209.}	136.251	0.871575	newly 50 weeks cumul	2740.	1819.99	{158., 8406.}	113.024	0.976671
35	newly 3 weeks spec	441.	47.06	{0., 107.}	91.932	2.13344	newly 3 weeks cumul	1805.	1365.73	{28., 3434.}	116.067	0.878156	newly 5 weeks spec	239.	33.58	{0., 77.}	93.2887	2.3488	newly 5 weeks cumul	2353.	1436.79	{31., 4277.}	104.698	0.903296	newly 57 weeks cumul	2899.	1615.05	{40., 4277.}	96.1645	0.88154
36	newly 3 weeks spec	507.	69.6	{0., 98.}	96.0197	2.38851	newly 3 weeks cumul	1570.	951.76	{263., 1313.}	69.2841	0.462988	newly 5 weeks spec	332.	5.95	{0., 12.}	98.2078	2.87942	newly 5 weeks cumul	2346.	978.36	{263., 1319.}	75.2796	0.610149	newly 53 weeks cumul	3516.	980.88	{263., 1319.}	80.0336	0.766954
37	newly 3 weeks spec	390.	214.19	{18., 613.}	102.403	0.842038	newly 3 weeks cumul	1418.	1056.81	{224., 2799.}	98.0007	0.56195	newly 5 weeks spec	278.	113.08	{1., 104.}	107.662	1.33721	newly 5 weeks cumul	2082.	1328.73	{247., 3805.}	98.8602	0.656071	newly 56 weeks cumul	2812.	1520.75	{255., 4066.}	96.708	0.731265
38	newly 3 weeks spec	545.	211.78	{19., 572.}	93.1193	0.922147	newly 3 weeks cumul	1921.	1180.96	{213., 2818.}	97.1692	0.66792	newly 5 weeks spec	367.	99.06	{3., 76.}	101.956	1.4121	newly 5 weeks cumul	2763.	1429.54	{227., 3578.}	96.1426	0.757475	newly 56 weeks cumul	3774.	1584.61	{234., 3815.}	93.3272	0.841867
39	newly 3 weeks spec	417.	42.16	{0., 49.}	93.6019	1.76548	newly 3 weeks cumul	1435.	594.93	{80., 595.}	93.5645	0.784912	newly 5 weeks spec	292.	4.23	{0., 11.}	98.5514	2.73028	newly 5 weeks cumul	2099.	613.12	{80., 607.}	93.09	0.926127	newly 52 weeks cumul	3353.	615.25	{80., 607.}	92.6624	1.11166
40	newly 3 weeks spec	501.	152.55	{22., 388.}	82.1737	0.878968	newly 3 weeks cumul	1605.	582.94	{163., 1136.}	77.924	0.704144	newly 5 weeks spec	314.	98.17	{3., 298.}	83.6975	1.03058	newly 5 weeks cumul	2336.	802.56	{214., 1774.}	79.5377	0.750198	newly 52 weeks cumul	3452.	1187.62	{274., 2793.}	79.9363	0.788207
41	newly 3 weeks spec	586.	229.6	{4., 307.}	109.215	1.41641	newly 3 weeks cumul	2370.	943.03	{146., 2249.}	91.0916	0.956712	newly 5 weeks spec	259.	52.42	{0., 155.}	94.556	2.06249	newly 5 weeks cumul	2995.	1110.5	{147., 3053.}	91.0584	0.998659	newly 60 weeks cumul	3338.	1177.69	{147., 3054.}	90.6702	1.02819
42	newly 3 weeks spec	574.	340.83	{0., 1386.}	96.8833	1.81861	newly 3 weeks cumul	2136.	1813.32	{78., 5286.}	97.236	0.768382	newly 5 weeks spec	240.	136.19	{0., 423.}	91.9542	1.67458	newly 5 weeks cumul	2747.	2169.88	{78., 6569.}	92.5643	0.794484	newly 51 weeks cumul	3203.	2466.82	{78., 6933.}	90.1461	0.797993
43	newly 3 weeks spec	451.	98.34	{0., 90.}	99.663	1.87811	newly 3 weeks cumul	1975.	733.06	{16., 746.}	102.383	1.17429	newly 5 weeks spec	196.	37.05	{0., 37.}	102.26	2.33353	newly 5 weeks cumul	2487.	826.48	{18., 1020.}	100.123	1.23294	newly 59 weeks cumul	2787.	935.67	{18., 1020.}	100.806	1.2668
44	newly 3 weeks spec	403.	73.71	{0., 96.}	95.5558	2.20844	newly 3 weeks cumul	1374.	1118.83	{216., 2659.}	81.0895	0.477125	newly 5 weeks spec	237.	18.69	{0., 8.}	100.181	2.70784	newly 5 weeks cumul	1901.	1179.64	{216., 3514.}	80.6891	0.580395	newly 53 weeks cumul	2836.	1190.49	{216., 3514.}	78.8142	0.7097
45	newly 3 weeks spec	487.	175.8	{21., 242.}	92.0903	0.91968	newly 3 weeks cumul	1897.	1069.23	{221., 1455.}	91.8297	0.667564	newly 5 weeks spec	273.	61.21	{2., 63.}	97.2491	1.32073	newly 5 weeks cumul	2573.	1230.14	{240., 1613.}	91.2981	0.737333	newly 56 weeks cumul	3089.	1326.78	{248., 1698.}	90.9446	0.782793
46	newly 3 weeks spec	405.	147.05	{0., 121.}	107.578	1.79333	newly 3 weeks cumul	1271.	1100.05	{68., 2749.}	127.71	0.858007	newly 5 weeks spec	319.	61.54	{0., 87.}	95.6552	1.94476	newly 5 weeks cumul	1984.	1258.39	{76., 3664.}	112.934	0.947807	newly 55 weeks cumul	2681.	1385.01	{80., 4781.}	103.989	1.0007
47	newly 3 weeks spec	463.	199.81	{13., 603.}	90.8402	0.884672	newly 3 weeks cumul	1416.	1087.76	{206., 2455.}	99.0692	0.574502	newly 5 weeks spec	259.	115.48	{1., 106.}	109.073	1.28425	newly 5 weeks cumul	2039.	1359.35	{222., 3963.}	98.2585	0.657301	newly 56 weeks cumul	2825.	1574.22	{224., 4615.}	96.1791	0.741694
48	newly 3 weeks spec	614.	174.54	{9., 334.}	76.1238	0.929675	newly 3 weeks cumul	1986.	1340.88	{160., 2183.}	56.7271	0.392721	newly 5 weeks spec	377.	63.83	{0., 156.}	83.9761	1.46581	newly 5 weeks cumul	2819.	1509.09	{164., 2383.}	60.9071	0.466936	newly 53 weeks cumul	3973.	1623.41	{164., 2393.}	67.811	0.56887
49	newly 3 weeks spec	412.	130.21	{0., 101.}	108.216	1.99709	newly 3 weeks cumul	1467.	1210.07	{54., 4210.}	123.529	0.827975	newly 5 weeks spec	214.	53.41	{0., 64.}	101.304	1.96775	newly 5 weeks cumul	2027.	1347.94	{69., 4210.}	114.187	0.885308	newly 55 weeks cumul	2605.	1461.01	{74., 4210.}	106.135	0.922111
50	newly 3 weeks spec	434.	212.92	{0., 579.}	105.829	1.46995	newly 3 weeks cumul	2003.	2114.81	{167., 9683.}	126.738	0.72326	newly 5 weeks spec	156.	60.04	{0., 134.}	98.6154	1.78863	newly 5 weeks cumul	2450.	2288.	{177., 11 047.}	118.079	0.751101	newly 60 weeks cumul	2707.	2369.93	{177., 11 324.}	113.229	0.76214
